# Intrahepatic Sarcomatoid Cholangiocarcinoma

**DOI:** 10.1155/2010/701476

**Published:** 2010-04-29

**Authors:** Shalini Malhotra, Joanna Wood, Talal Mansy, Rajeev Singh, Abed Zaitoun, Srinivasan Madhusudan

**Affiliations:** ^1^Department of Histopathology, Nottingham University Hospitals, Queens Medical Centre Campus, University of Nottingham, NG7 2UH Nottingham, UK; ^2^Academic Unit of Oncology, Nottingham University Hospitals, City Hospital Campus, University of Nottingham, NG5 1PB Nottingham, UK; ^3^Department of Radiology, Derby City General Hospital, DE22 3NE Derby, UK

## Abstract

Intrahepatic sarcomatoid cholangiocarcinoma is a rare but an aggressive variant of cholangiocarcinoma with a very poor prognosis. We report the first caucasian patient who presented with a rapidly enlarging liver mass requiring hepatic resection. Detailed histopathological analyses including immunohistochemistry and electron microscopy confirmed sarcomatoid cholangiocarcinoma. The patient had early onset disease recurrence within 5 weeks of surgery. Here we demonstrate that combination chemotherapy with gemcitabine and cisplatin is a potential treatment option in patients with advanced sarcomatous cholangiocarcinoma. The patient achieved sustained partial remission with combination chemotherapy and remains alive and well more than 29 months since initial surgery.

## 1. Introduction

Sarcomatoid changes of epithelial neoplasms are rare but found occasionally in renal cell carcinoma, squamous cell carcinoma (involving the lungs, oesophagus, and skin), and adenocarcinoma of the lung [1]. In the liver, sarcomatoid transformation has been reported in about 3.9 to 9.4% of hepatocellular carcinomas at autopsy and in about 4.5% of cholangiocarcinomas [2]. It has been reported that prognosis for intrahepatic sarcomatous cholangiocarcinoma is worse than that for a conventional intrahepatic cholangiocarcinoma. The factors that account for the poor prognosis may be ascribed to the remarkable intrahepatic development, especially the high potential of the sarcomatous component to metastasize. Vascular invasion has been shown to be more frequently present if there is a sarcomatous component [3]. Here we report a case of intrahepatic sarcomatoid cholangiocarcinoma in a caucasian patient and review the literature.

## 2. Case Report

**Figure 1:**
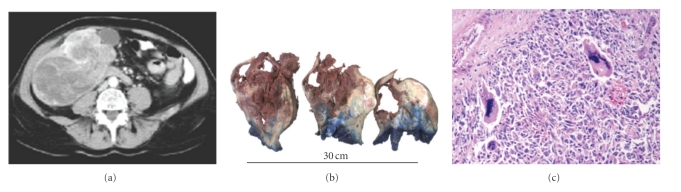
Radiological and histopathological features are shown here. Figure 1(a): preoperative CT-scan, Figure 1(b): gross histopathology and Figure 1(c): microscopy features. See text for details.

A 60-year-old Caucasian woman presented with an acute onset right upper abdominal pain and a rapidly enlarging upper abdominal mass. She had a previous history of resected early-stage melanoma. Clinical examination revealed tender hepatomegaly, and a contrasted computed tomography (CT) scan of the thorax, abdomen, and pelvis showed a large solitary heterogeneous mass in segment V1 of the liver with evidence of intratumoural bleeding (Figure 1(a)). She proceeded to laparotomy and at operation a large liver mass was evident and the tumour was found to be grossly adherent to a number of intraabdominal organs with evidence of tumour perforation and intraabdominal spillage. A lateral segmentectomy of the liver along with cholecystectomy was performed and she made an uneventful postoperative recovery.

The tumour measured 15 × 11 × 20 cm and cut section revealed massive necrosis and haemorrhage consistent with the preoperative CT scan (Figure 1(b)). The tumour extended to the liver capsule and to within 2 cm of the margin of excision. There was extensive vascular and perineural invasion. Histological examination of the tumour showed a malignant biphasic neoplasm consisting of a moderately differentiated adenocarcinoma intermingled with a malignant mesenchymal component (Figure 1(c)). The latter consisted of pleomorphic spindle cells arranged in sheets intermixed with bizarre multinucleate osteoclast like giant cells. Reams of osteoid surrounded by osteoblasts were also characteristically seen. The sarcomatous component made up to 70% of the whole tumour. The adenocarcinoma cells showed apparent mucus production as demonstrated by PAS and alcian blue staining. They neither displayed trabecular arrangement nor bile production, which are characteristics of a hepatocellular carcinoma. Immunohistochemical study of carcinomatous component revealed positive staining for Cam5.2, EMA, AE1/AE3, CK7, CK19, and CEA and negative staining for HePAR1. This immunoprofile was in keeping with cholangiocarcinoma. The sarcomatous component was positive for vimentin but negative for epithelial markers. S100, HMB45, MELAN A, desmin, inhibin, and CD117 were negative in both components. Both MiB1 and p 53 showed strong positivity in both epithelial and sarcomatous component (>80%). Electron microscopy showed presence of basement membrane and microvilli in the tumour cells which excluded their hepatocyte origin. It also demonstrated presence of tight junctions and desmosomes in the sarcomatous component indicating their epithelial origin. Based on histological, immunohistochemical and electron microscopy findings, a diagnosis of sarcomatoid cholangiocarcinoma was made and the patient was referred for consideration of adjuvant chemotherapy.

**Figure 2:**
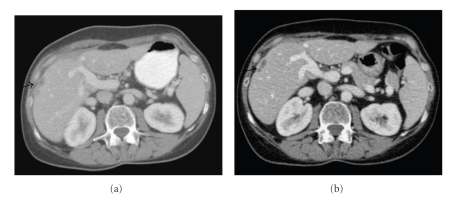
Postoperative CT-scan showing early disease recurrence (indicated with an arrow) within the liver (Figure 2(a)). Partial response after completion of chemotherapy is shown in Figure 2(b).

A postoperative contrasted CT scan at 5 weeks confirmed rapid onset disease recurrence with multiple peritoneal deposits and multifocal liver metastasis (Figure 2(a)). Patient was started on gemcitabine (1000 mg per m^2^ on days 1 and 8) and cisplatin (60 mg per m^2^ on day 1) chemotherapy in a 21-day cycle which she tolerated well with minimal side effects. Patient received a total of six cycles of chemotherapy and a restaging CT scan at the end of 6 cycles confirmed partial response (Figure 2(b)). Apart from grade 1 nausea that settled with antisickness medications, the patient tolerated chemotherapy very well. The patient reported improvement in appetite, weight gain, and resolution of abdominal swelling during chemotherapy. Following completion of treatment, she was started on clinical follow-up. A reassessment CT scan after 3 months of follow up showed further marginal disease size reduction. She continued under regular clinical follow-up until 10 months later; she developed further disease progression was rechallenged with gemcitabine and cisplatin at the same doses. A reassessment CT scan after chemotherapy showed partial response. She remains well and is now 29 months post initial presentation.

## 3. Discussion

Sarcomatoid cholangiocarcinoma is an uncommon variant of intrahepatic cholangiocarcinoma and in the previously reported series, the sarcomatoid component exhibited mainly spindle cell morphology. To the best of our knowledge osteoid differentiation and osteosarcoma like features in a sarcomatoid cholangiocarcinoma are very rare. Here, we describe the first case of such an entity in a Caucasian patient along with ultrastructural features. Sarcomatoid transformation has been reported to occur in a hepatocellular carcinoma previously treated with transcatheter arterial chemo embolisation regimes [4]. However, there have been no reports concerning the relationship between sarcomatoid cholangiocarcinoma and anticancer therapy. It is therefore reasonable to consider that sarcomatoid transformation is a natural course of tumour progression as far as a cholangiocarcinoma is concerned [2]. Most of the previously reported cases of sarcomatoid cholangiocarcinoma showed cytokeratin expression in both carcinomatous and sarcomatous components [2]. In our case, the sarcomatous component was negative for cytokeratins. This may be related to possible anaplastic dedifferentiation of tumour cells during sarcomatous transformation as occurred in 2 out of 7 cases reported by Nakajima et al. [5]. However we did confirm the epithelial derivation of the sarcomatous component in our case by electron microscopy. Though controversy has surrounded the histogenesis of sarcomatoid cholangiocarcinoma, we favour the concept of metaplastic transformation as has been well accepted for similar tumours of other organs [6]. 

Aggressive biology is highlighted by the fact that our patient had an early onset rapid recurrence of disease within a few weeks of surgery. The rapid recurrence is also indicative of early microscopic metastatic seedlings prior to surgery. In view of widespread recurrence, further surgical intervention was not performed and the patient proceeded to palliative chemotherapy. Kaibori et al. reviewed sixteen cases of sarcomatous cholangiocarcinomas previously reported in the literature. Seven patients had not had surgery and all had died within five months of diagnosis. Nine patients had liver resection but four of these patients died within seven months of surgery [7]. The survival rates of patients with surgically resected disease were significantly higher than in patients without resection [7, 8]. There is however, no information available about the optimal adjunctive treatment after initial surgical resection. Table 1 summarises clinicopathological characteristics of patients with sarcomatoid cholangiocarcinoma previously published in the literature. To the best of our knowledge, there is also no published report in Caucasian patients demonstrating response and survival benefit to systemic chemotherapy. A combination of gemcitabine and cisplatin has been shown to produce antitumour responses in metastatic biliary tract cancer [9] and sarcoma [10]. Based on this evidence, our patient received this combination which was well tolerated. Patient achieved partial response after 6 cycles of chemotherapy confirming the clinical activity of this combination in this disease. In addition, patient demonstrated improvements in clinical symptoms during chemotherapy which was sustained after completion and at follow-up. The patient remains alive and well sixteen months since initial presentation. Given the fact that she presented with rapidly progressive liver mass and a rapid onset disease recurrence, it is very likely that this combination chemotherapy resulted in a survival benefit for this patient associated with improvement in quality of life. 

In conclusion, we have demonstrated that combination chemotherapy with gemcitabine and cisplatin is a potential treatment option in patients with advanced sarcomatous cholangiocarcinoma. Review of previous reports suggests that radical surgery is the treatment of choice in those with limited disease. However, the role of adjuvant chemotherapy is unknown. As the prognosis of this tumour type is very poor, and the amount of reported clinical data is limited, our report raises the need to consider the potential benefit of adjuvant gemcitabine/cisplatin chemotherapy in patients with resected intrahepatic sarcomatoid cholangiocarcinoma.

## Figures and Tables

**Table 1:** Clinicopathological features of previously published patients with sarcomatoid cholangiocarcinoma.

	no. of patients	Sarcomatoid component	Treatment	Survival
Kaibori et al. [7]	1	Pleomorphic and spindle cells	Surgery	Patient died ≤3 months after surgery with recurrence
Sumiyoshi et al. [8]	1	Spindle cell	None	Patient died within 2 months after diagnosis
Sato et al. [2]	1	Round cell variant	None	Patient died within 3 months of diagnosis
Nakajima et al. [5]	7	Spindle cell (5)	Surgery (2)	- Patient died ≤3 months after diagnosis (4)
		Pleomorphic and spindle cell (2)	TAE (2)	- Patient died 3–6 months after diagnosis (2)
			Chemotherapy* (1)	- Patient alive beyond 6 months (1)
			None (2)	
Aishima et al. [3]	7	Not available	Surgery (7)	5 patients died of disease recurrence. 3 year survival (17.9%)

TAE (Trans arterial chemoembolisation), *details of chemotherapy not available.
